# Myrtenol Reduces Orofacial Nociception and Inflammation in Mice Through p38-MAPK and Cytokine Inhibition

**DOI:** 10.3389/fphar.2022.910219

**Published:** 2022-05-30

**Authors:** Janaíne P. Oliveira, Fabíula F. Abreu, José Marcos M. Bispo, Anderson R. A. Cerqueira, José Ronaldo dos Santos, Cristiane B. Correa, Soraia K. P. Costa, Enilton A. Camargo

**Affiliations:** ^1^ Graduate Program in Physiological Sciences, Federal University of Sergipe, São Cristóvão, Brazil; ^2^ Department of Pharmacology, Institute of Biomedical Sciences, University of São Paulo, São Paulo, Brazil; ^3^ Department of Biosciences, Federal University of Sergipe, Itabaiana, Brazil; ^4^ Department of Morphology, Federal University of Sergipe, São Cristóvão, Brazil; ^5^ Department of Physiology, Federal University of Sergipe, São Cristóvão, Brazil

**Keywords:** orofacial pain, temporomandibular disorder, cytokine, mitogen-activated protein kinase, terpene, myrtenol

## Abstract

Orofacial pain is one of the commonest and most complex complaints in dentistry, greatly impairing life quality. Preclinical studies using monoterpenes have shown pharmacological potential to treat painful conditions, but the reports of the effects of myrtenol on orofacial pain and inflammation are scarce. The aim of this study was to evaluate the effect of myrtenol in experimental models of orofacial pain and inflammation. Orofacial nociceptive behavior and the immunoreactivity of the phosphorylated p38 (P-p38)-MAPK in trigeminal ganglia (TG) and spinal trigeminal subnucleus caudalis (STSC) were determined after the injection of formalin in the upper lip of male Swiss mice pretreated with myrtenol (12.5 and 25 mg/kg, i.p.) or vehicle. Orofacial inflammation was induced by the injection of carrageenan (CGN) in the masseter muscle of mice pretreated with myrtenol (25 and 50 mg/kg, i.p.) or its vehicle (0.02% Tween 80 in saline). Myeloperoxidase (MPO) activity and histopathological changes in the masseter muscle and interleukin (IL)-1β levels in the TG and STSC were measured. The increase in face-rubbing behavior time induced by formalin and P-p38-MAPK immunostaining in trigeminal ganglia were significantly reduced by myrtenol treatment (12.5 and 25 mg/kg). Likewise, increased MPO activity and inflammatory histological scores in masseter muscle, as well as augmented levels of IL-1β in the TG AND STSC, observed after CGN injection, were significantly decreased by myrtenol (25 and 50 mg/kg). Myrtenol has potential to treat orofacial inflammation and pain, which is partially related to IL-1β levels in the trigeminal pathway and p38-MAPK modulation in trigeminal ganglia.

## Introduction

Orofacial pain affects the head and neck regions and can be associated with inflammation. Temporomandibular disorders are mainly conditions of non-odontogenic origin related to orofacial pain (Hargreaves, 2011; Dym and Israel, 2012). They are complex pathologies in which myofascial pain has high prevalence (Fernandes et al., 2018).

The clinical management of orofacial pain is still a challenge. Besides the administration of drugs like non-steroidal anti-inflammatories, corticosteroids, anticonvulsants, and tricyclic antidepressants, behavioral (self-education), physiological and physical therapies (e.g., physiotherapy and acupuncture) also are potentially useful for the management of this condition (Shephard et al., 2014; Aggarwal et al., 2019). However, in many patients these treatments still lack efficacy or cause a wide range of undesired side effects.

It is clearly necessary to find new alternative approaches to reduce symptomatology and improve life quality of patients with orofacial pain (Aggarwal et al., 2019). In this respect, natural products possess therapeutic potential for the treatment of many diseases and conditions, including inflammation and pain (Dewick, 2009; Guimarães et al., 2013). Myrtenol is one these natural products, belonging to the class of terpenes. It has anti-inflammatory and antinociceptive properties (Silva et al., 2014; Gomes et al., 2017), but there is no information about its possible effects on painful orofacial disorders.

Evidence shows that other terpenes can reduce nociception in orofacial pain (Oliveira et al., 2020). Furthermore, myrtenol has been found to have antinociceptive action in models of acetic acid-induced abdominal contortions and formalin-, glutamate- and capsaicin-induced nociception in mouse paws (Silva et al., 2014). Thus, we evaluated the effect of myrtenol in experimental orofacial models of pain and inflammation and investigated possible mechanisms of action involved, to expand the possibilities of using this terpenoid substance to treat pain in the orofacial region.

## Materials and Methods

### Animals

Male Swiss mice (25–35 g, 2–3 months of age) were obtained from the animal center of Federal University of Sergipe. The animals were kept at 21–23°C with free access to food and water under a 12-h light/dark cycle. All animals had the same environmental conditions and basal characteristics. They were maintained conscious or anesthetized with intraperitoneal (i.p.) ketamine (80 mg/kg) and xylazine (8 mg/kg) depending on the experimental protocol. All experiments were conducted in agreement with the guidelines of the Brazilian College of Animal Experimentation and the National Institutes of Health Guidelines and were approved by the Ethics Committee on Animal Use of our institution (CEPA 19/18). Allocation and group separation were performed randomly by using the setting available at *random.org*. At the end of the experiments, the animals were euthanized by overdose of anesthesia.

### Orofacial Formalin Test

Orofacial nociception was assessed by the formalin test. For this, formalin was injected (20 μL; 2%) in the right upper lip with a 27 G needle (Quintans-Júnior et al., 2010). Thirty minutes before orofacial nociception induction, animals (n = 6 per group) were treated with myrtenol (12.5 or 25 mg/kg, i.p.), vehicle (0.9% saline with 0.02% Tween 80, i.p.) or morphine (an opioid analgesic used as positive control, 5 mg/kg). The quantification of nociception was measured by the time (in seconds) of face rubbing in the first (from 0 to 5 min) and the second phases (from 15 to 40 min) after formalin injection. For this, animals were maintained in mirror boxes and their orofacial movements were recorded by a camcorder (Samsung DV Mod. SC-D382) for 40 min. These records were analyzed by an investigator blinded to the group identity.

We performed immunohistochemistry analyses to investigate the participation of the p38-MAPK pathway in the action mechanism of myrtenol. Immediately after the formalin test, animals were deeply anesthetized by ketamine and xylazine injection (i.p.) and perfused with PBS (pH 7.4), followed by paraformaldehyde (4.0%) in phosphate buffer (0.1 M, pH 7.4). The brain and trigeminal ganglia ipsilateral to the formalin injection site were removed from the skull, postfixed in the same fixative solution for 24 h, and transferred to a solution containing sucrose (30% in 0.1 M PBS). Each sample was serially cut in the coronal plane into 30 µm thick sections with a cryostat microtome (Leica, Germany). The sections were placed in sheets. Primary antibodies for phosphorylated p38 (P-p38)-MAPK (1:1,000, Cell Signaling Technology) were incubated overnight (for 18 h) at 4°C. Afterwards, the sections were incubated with the biotinylated goat anti-rabbit secondary antibodies (1:1,000; Sigma Chemical Company) for 2 h at room temperature, washed, and incubated with avidin–biotin-peroxidase solution (ABC Elite kit, Vector Labs, Burlingame, United States) for 90 min. The reaction was developed by the addition of diaminobenzidine tetrahydrochloride (DAB; Sigma, United States) and H_2_O_2_ (0.01%). Sections were examined under brightfield illumination (Olympus Microscope, BX-41), images were captured using a CCD camera (Nikon Eclipse Ci-S), and the spinal trigeminal subnucleus caudalis and trigeminal ganglia locations were determined using the atlas of Paxinos and Franklin (2001). The P-p38-MAPK positive cell count was performed for the whole extension of the evaluated regions within each section, using ImageJ (Version 1.46i, NIH). In each section, four fields evenly distributed throughout the areas of interest were analyzed by an investigator unaware of the experimental groups. Finally, all values were normalized considering the control group.

### Orofacial Inflammation Induced by Carrageenan in Masseter Muscle

Orofacial inflammation was induced by injecting carrageenan (3%, 20 μL, n = 6–8 per group, Bagüés et al., 2017) into the right masseter muscle of anesthetized mice (3% isoflurane). The control group received saline solution (0.9%, 20 μL). The injection site was determined by masseter muscle palpation between the mandible and zygomatic bone (Bagüés et al., 2017). Thirty minutes before induction, animals were intraperitoneally treated with myrtenol (25 or 50 mg/kg, Sigma-Aldrich, St. Louis, MO, United States), indomethacin (a non-steroidal anti-inflammatory, 10 mg/kg) or vehicle (0.9% saline with 0.02% Tween 80). After 6 hours, animals were euthanized by isoflurane overdose followed by cervical dislocation. Then the ipsilateral masseter muscle, trigeminal ganglia and spinal trigeminal subnucleus caudalis were collected, washed in PBS and immediately frozen for posterior analyses. All the treatments were performed by a researcher blinded to the group identification.

Myeloperoxidase (MPO) activity was determined in masseter muscle homogenates as previously described (Souza et al., 2018). Results were expressed as units of MPO per site per mg of tissue. A unit of MPO was considered to be the amount of enzyme that degraded 1 mmol of hydrogen peroxide/min (Bradley et al., 1982).

For histological analysis, masseter muscle samples were processed according to routine histological techniques. Transversal sections with 5 µm thickness were stained with hematoxylin and eosin and analyzed by light microscopy to determine the intensity of tissue alterations and leukocyte infiltration in muscle tissue. Scores were classified from 0 to 4 by a researcher blinded to group identity, where: 0 = absence of alterations; 1 = rare alterations; 2 = moderate alterations; 3 = intense alterations; 4 = severe alterations. These scores were used to evaluate edema, necrosis, and inflammatory infiltrate. The results were expressed as the sum of individual scores.

The levels of IL-1β were measured in ipsilateral trigeminal ganglia and spinal trigeminal subnucleus caudalis. Briefly, these tissues, obtained from 8 animals, were randomly pooled with 2 animals for each sample (resulting in n = 4 measurements) and homogenized in a solution of phosphate-buffered saline (pH 7.2) with Tween 20 (0.05%), phenylmethylsulfonyl fluoride (0.1 mM), benzethonium chloride (0.1 mM), EDTA (10 mM) and aprotinin A (2 ng/ml). Homogenates were centrifuged at 8,000*xg* for 10 min at 4°C and supernatants were collected. IL-1β levels were evaluated by a commercial ELISA kit according to the manufacturer’s instructions (R&D Systems). Results were expressed in pg of cytokine/mg of protein. The protein quantity of each sample was measured by the Bradford method.

### Statistical Analysis

The results are expressed as means ± SEM. For the statistical evaluation, data were analyzed by the Shapiro-Wilk normality test and no impediment to the use of parametric analysis was found. Thus, we performed one-way analysis of variance (ANOVA) followed by the Tukey test. *p*-values lower than 0.05 were considered significant.

## Results

### Effect of Myrtenol on Formalin-Induced Orofacial Nociception and p38-MAPK Pathway

**FIGURE 1:**
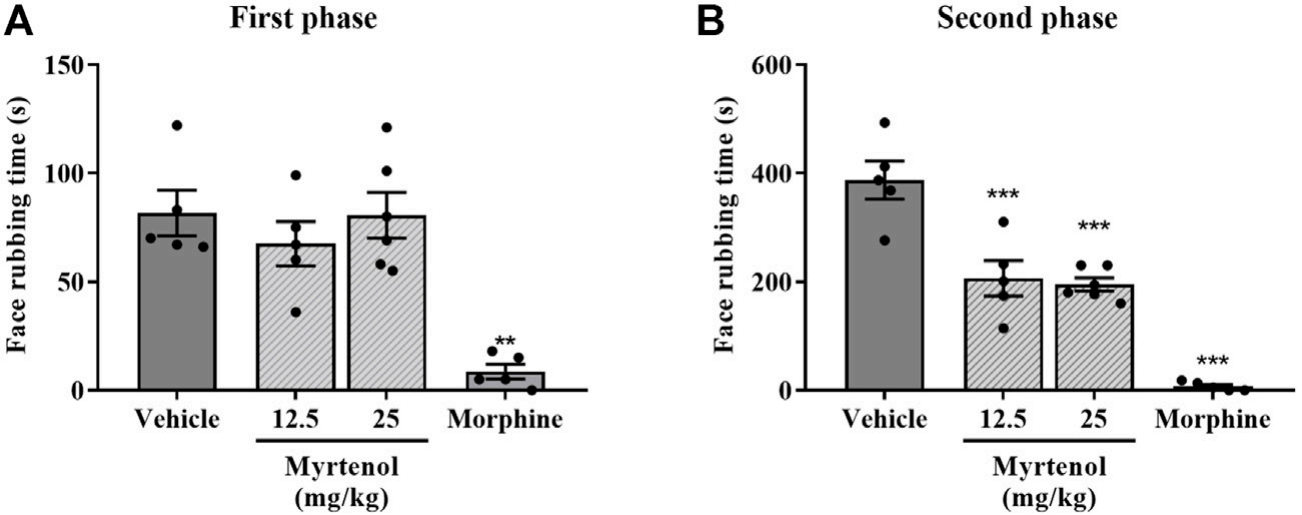
Effect of myrtenol pretreatment on formalin-induced orofacial nociception. Nociceptive behavior was measured as the face rubbing time in seconds (n = 5–6). **(A)**: first phase of formalin test, **(B)**: second phase of formalin test. ANOVA [F (3,17) = 13.18, *p* = 0.001 and F (3,17) = 40.93, *p* < 0.001, respectively] followed by Tukey´s post-test, **p < 0.01 or ***p < 0.001 vs. vehicle.

Pretreatment with myrtenol at 12.5 and 25 mg/kg reduced face-rubbing time in the second phase, but not in the first phase, of the formalin test in comparison with the vehicle group (*p* < 0.05 and *p* < 0.01, respectively; Figure 1). Pretreatment with morphine reduced face-rubbing time in both phases (*p* < 0.01 and *p* < 0.001 for the first and the second phase, respectively) in comparison with the vehicle group (Figure 1).

**FIGURE 2:**
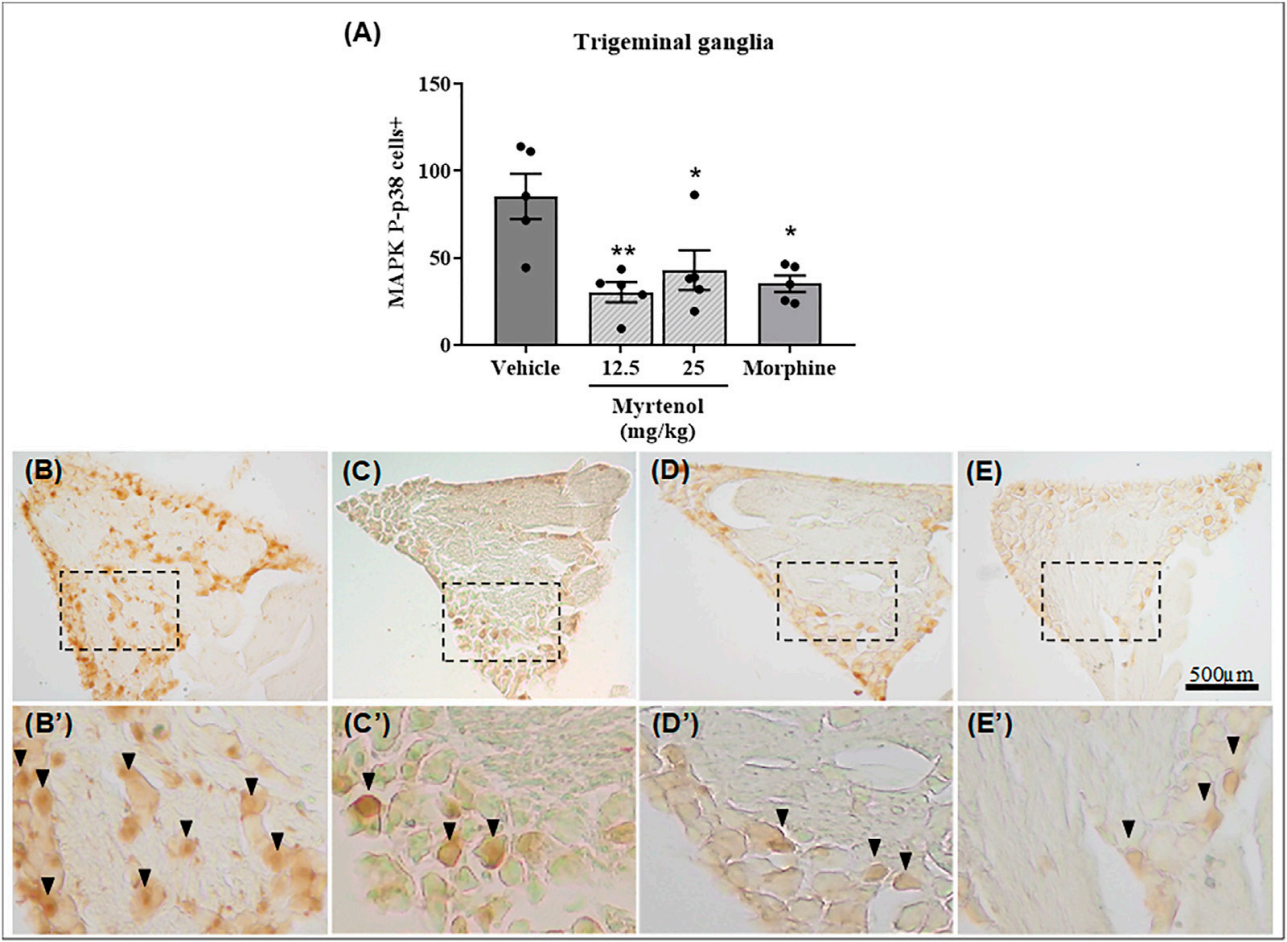
Myrtenol reduces phosphorylated p38-MAPK staining in trigeminal ganglia of mice submitted to orofacial formalin test. **(A)**: Each bar represents the mean ± SEM of the number of the positive cells by phosphorylated p38-MAPK staining (n = 5). ANOVA [F (3,16) = 7.19, *p* = 0.0028] followed by Tukey´s test, *p < 0.05 or **p < 0.01 vs. vehicle group. **(B–E)**: representative images (40 x) of vehicle + formalin **(B)**, myrtenol (12.5 mg/kg) + formalin **(C)**, myrtenol (25 mg/kg) + formalin **(D)** and morphine (5 mg/kg) + formalin **(E)** groups. Panels B′-E’: higher magnification (100x) of dotted areas from **(B–E)**, respectively.

By using immunohistochemistry analysis, we detected a lower number of positively stained cells for P-p38 MAPK in the trigeminal ganglia (*p* < 0.05 and *p* < 0.001, respectively) of animals pretreated with myrtenol at the doses of 12.5 and 25 mg/kg in comparison with the vehicle group (Figure 2). We did not find alteration in positive cell counts for P-p38-MAPK in the spinal trigeminal subnucleus caudalis after nociception induction in all the groups evaluated (data not shown).

### Effect of Myrtenol on Carrageenan-Induced Orofacial Inflammation in Masseter Muscle and Cytokine Production in Trigeminal Ganglia and Spinal Trigeminal Subnucleus Caudalis

**FIGURE 3:**
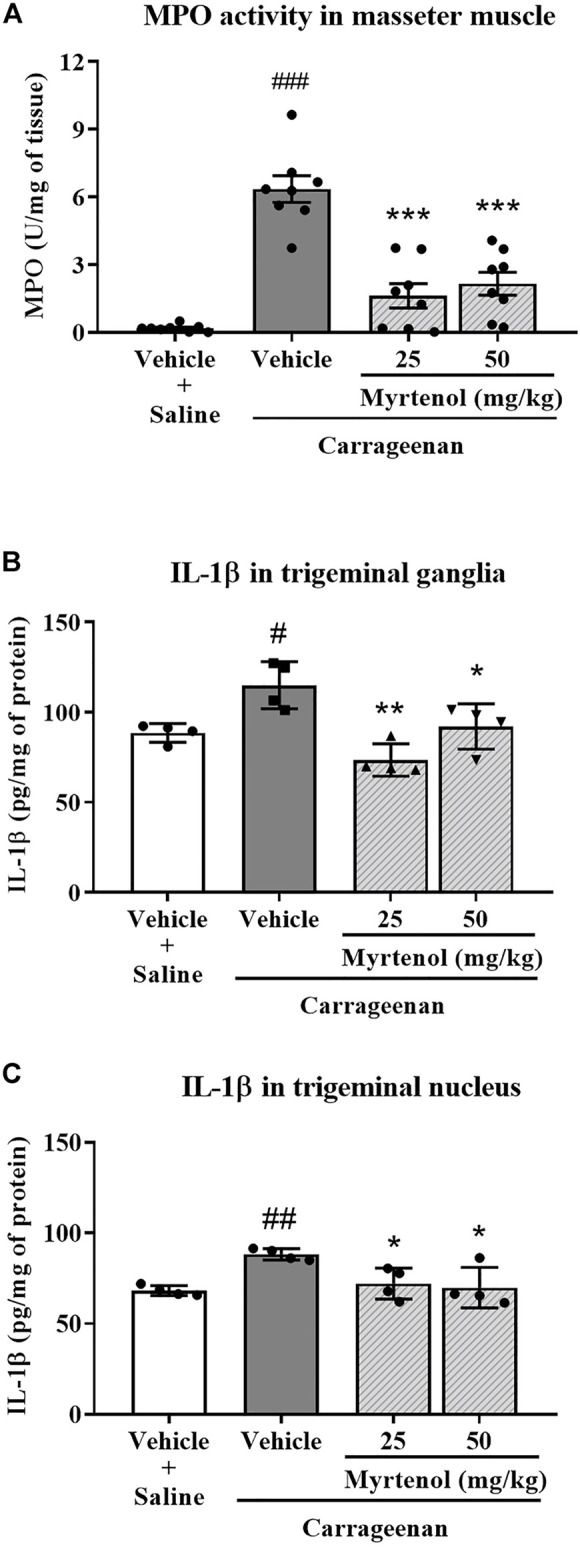
Myrtenol decreases myeloperoxidase (MPO) activity in masseter muscle and IL-1β levels in trigeminal ganglia and spinal trigeminal subnucleus caudalis in carrageenan-induced orofacial inflammation in mice. **(A)**: MPO activity (n = 8) in masseter muscle. **(B,C)** (n = 4 each): IL-1β levels in trigeminal ganglia and spinal trigeminal subnucleus caudalis respectively. ANOVA [F (3,28) = 24.51, *p* < 0.001; F (3,12) = 10.81, *p* = 0.001 and F (3,12) = 6.79, *p* = 0.0081, respectively] followed by Tukey test, ^#^ *p* < 0.05, ^##^ *p* < 0.01 or ^###^ *p* < 0.001 vs. saline + vehicle; *p < 0.05, **p < 0.01 or ***p < 0.001 vs. carrageenan + vehicle.

To investigate the effects of myrtenol in the carrageenan-induced orofacial inflammation model, we used doses of 25 mg/kg (the same as in the formalin model) and 50 mg/kg of this monoterpene. In groups pretreated with myrtenol at these doses, we found lower MPO activity (*p* < 0.001 each dose) in comparison to the vehicle group. The same was noted for pretreatment with indomethacin (*p* < 0.0001 vs. vehicle group; Figure 3A).

We quantified the pro-inflammatory cytokine IL-1β levels in trigeminal ganglia and spinal trigeminal subnucleus caudalis of animals. Six hours after carrageenan injection, pretreatment with myrtenol reduced IL-1β levels both in trigeminal ganglia (*p* < 0.01–25 mg/kg and *p* < 0.05–50 mg/kg, Figure 3B) and in the spinal trigeminal subnucleus caudalis (*p* < 0.05 for each dose, Figure 3C) in comparison with the vehicle-treated group.

**FIGURE 4:**
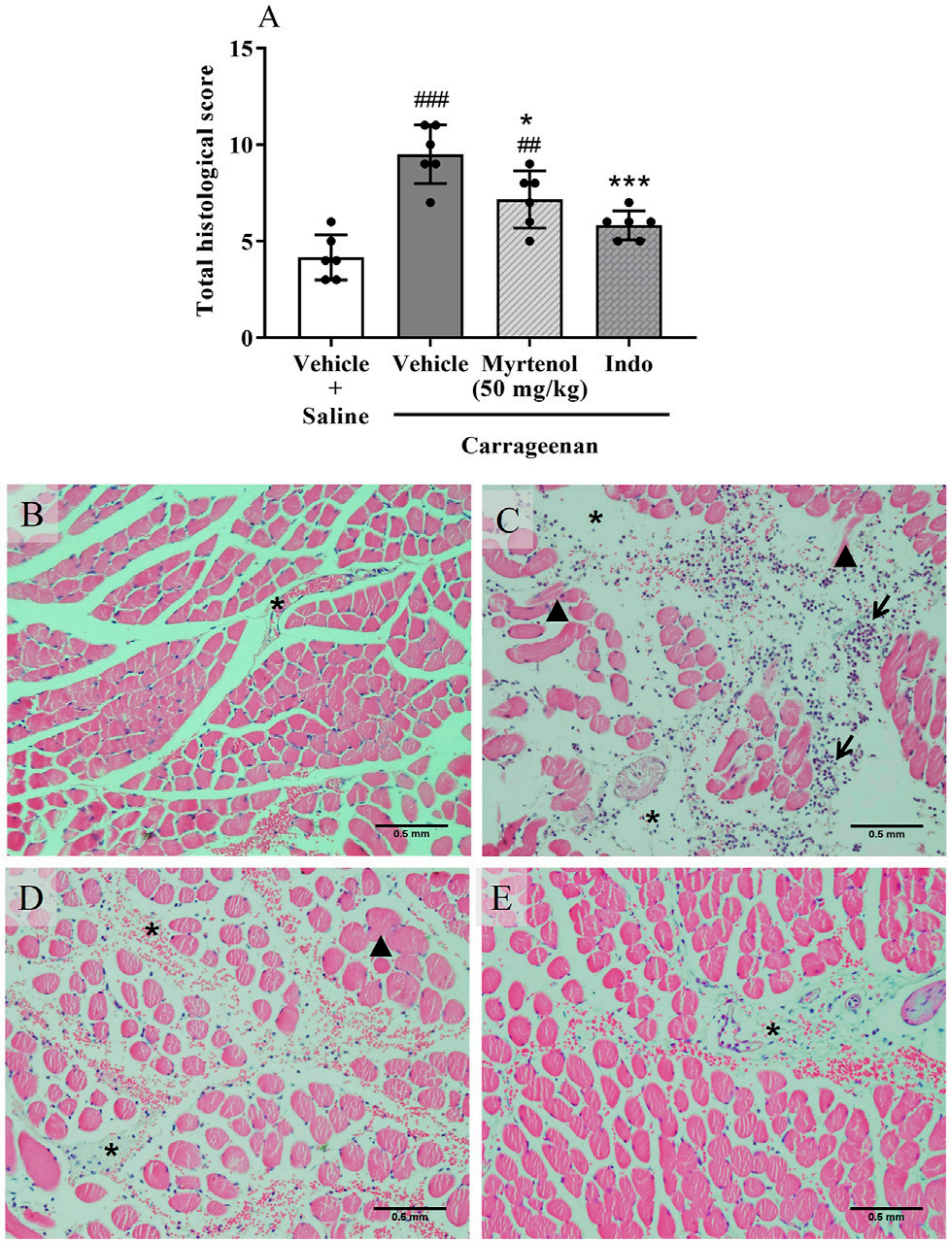
Myrtenol ameliorates carrageenan-induced histopathological changes in mouse masseter muscle. **(A–D)**: representative images (40 x) of vehicle + saline **(A)**, vehicle + carrageenan **(B)**, myrtenol (50 mg/kg) + carrageenan **(C)** and indomethacin + carrageenan **(D)** groups, respectively. Asterisk: edema; arrow: inflammatory infiltrate; arrowhead: necrosis. **(E)**: sum of scores (n = 6). ANOVA [F (3,20) = 19.03, *p* < 0.001] followed by Tukey test, ^##^ *p* < 0.01 and ^###^ *p* < 0.001 vs. saline + vehicle, *p < 0.05, ***p < 0.01 vs. carrageenan + vehicle.

Histological analysis of masseter muscle sections was also performed to further investigate the anti-inflammatory effect. In the representative image of the saline group, we observed rare or moderate presence of edema, leukocyte infiltrate and necrosis of muscle fibers (Figure 4B). In sections of the carrageenan group, we detected intense or severe presence of these parameters (Figure 4B). In the group pretreated with myrtenol (50 mg/kg; Figure 4C), we observed moderate or intense presence of edema, inflammatory infiltrate, and necrosis. Similar alterations were observed in animals pretreated with indomethacin (Figure 4D). An increased total score was verified in the carrageenan group in comparison with the saline group (*p* < 0.01, Figure 4E). In contrast, in the group pretreated with myrtenol, we observed a partial reduction of the total score in comparison with the carrageenan group (*p* < 0.05), like the alterations found in the group pretreated with indomethacin (*p* < 0.01, Figure 4E).

## Discussion

In this study, we present for the first-time data about the protective effect of myrtenol on nociception and inflammation in the orofacial region with modulation of the trigeminal pathway.

We first observed that myrtenol reduced face rubbing behavior after formalin challenge. In the first phase of this test, activation occurs of nociceptive terminals, which convey sensorial information to the trigeminal nociceptive system. In the second phase, inflammatory mediators are generated that enhance nerve fiber stimulation (Raboisson and Dallel, 2004). The fact that myrtenol affected the nociception in the second phase but not on the first phase of the formalin test suggests this response is related to the anti-inflammatory action of this compound. Silva and coworkers (2014) observed a similar effect in formalin-induced nociception in mouse paws by using 75 mg/kg of myrtenol, which is a much higher dose than ours (25 mg/kg), but they also found that myrtenol pretreatment reduced the nociceptive behavior only in the second phase. Furthermore, treatment with myrtenol did not affect nociception in the hot plate test (Silva et al., 2014), a method traditionally used to evaluate the central component of nociception (Gunn et al., 2011). A recent study showed that treatment with myrtenol complexed with β-cyclodextrin (25, 50, 100 mg/kg) reduced paw mechanical hyperalgesia induced by acid saline injection in the gastrocnemius muscle, a model of chronic widespread pain that is considered to mimic aspects of fibromyalgia in humans (Heimfarth et al., 2020). Together these studies, our data support the antinociceptive effect of myrtenol.

Of importance, other authors have reported that treatment with myrtenol at doses up to 75 mg/kg neither altered motor performance in the rota-rod test (Silva et al., 2014) nor modified spontaneous motor activity in open field testing (Gomes et al., 2017), which minimize the possibility that the inhibition of the nociceptive behavior of mice caused by myrtenol was biased by effects like muscular relaxation or central depression.

Many signaling pathways can contribute to the antinociception observed. We measured the phosphorylated p38-MAPK in trigeminal ganglia and spinal trigeminal subnucleus caudalis after orofacial formalin testing and found that the activation of this kinase was reduced by treatment with myrtenol only in trigeminal ganglia. This finding is important because the MAPK pathway is a crucial component of the peripheral and central nociceptive sensitization in neuropathic, chronic, and inflammatory pain (Ji et al., 2013; Edelmayer et al., 2014). The activation of this pathway occurs in many nociceptive animal models, including the formalin test in rodent paws and lips (Bastos et al., 2007; Li et al., 2010; Tan et al., 2012). It can also occur early in nociception pathways, more particularly in primary sensory neurons or satellite cells of dorsal root ganglia or trigeminal ganglia, as well as in second-order neurons located in the dorsal horn of the spinal medulla and trigeminal spinal trigeminal subnucleus caudalis to supraspinal neurons of cortico-thalamic regions (Edelmayer et al., 2014; Kiyomoto et al., 2015).

Considering the results of the second phase of formalin test, we tested the hypothesis that myrtenol could also affect acute orofacial inflammation induced by carrageenan. Interestingly, pretreatment with myrtenol reduced MPO activity in masseter muscle, an indication of reduction of neutrophils in this tissue. This hypothesis was supported by the decrease of edema, necrosis, and leukocyte infiltrate observed by histological analysis in the masseter muscle of animals treated with myrtenol. In agreement with these results, Silva and coworkers (2014) reported that treatment with myrtenol (75 mg/kg) reduced MPO activity in paws injected with carrageenan. Viana et al. (2016) also reported that myrtenol (50 mg/kg) reduced MPO activity in gastric lesions induced by ethanol in mice. Furthermore, Gomes and coworkers (2017) observed that incubation with myrtenol reduced cellular migration of human neutrophils stimulated with n-formylmethionine-leucyl-phenylalanine.

Carrageenan injection in the masseter muscle increased IL-1β levels in trigeminal ganglia and spinal trigeminal subnucleus caudalis, suggesting that neuroinflammation is triggered in this model of orofacial inflammation and might contribute to sensitization of the sensory fibers involved. Similar to our findings, other authors have reported that peripheral injection with pro-inflammatory agents is capable of triggering neuroinflammation. For example, carrageenan injection in mouse paws increased IL-1β levels in the spinal cord (Lu et al., 2013; Choi et al., 2015). In the orofacial region, masseter muscle inflammation induced by CFA increased IL-1β expression in the trigeminal spinal subnucleus caudalis (Guo et al., 2007).

Our data showed that treatment with myrtenol reduced levels of IL-1β in trigeminal ganglia and spinal subnucleus caudalis. These findings suggest that this compound can directly or indirectly modulate central nociceptive pathways due to inhibition of pro-inflammatory cytokine production. Previous studies have shown that myrtenol inhibits pro-inflammatory cytokines, but only peripherally. Silva and coworkers (2014) reported that pretreatment with myrtenol reduced IL-1β levels in peritoneal lavage of mice submitted to carrageenan-induced peritonitis. In the same way, daily treatment with myrtenol for a week reduced IL-1β, TNF-α and interferon-γ in bronchoalveolar lavage of rats challenged with ovalbumin (Bejeshk et al., 2019). In the model of gastric ulcer induced by acetic acid, myrtenol treatment for 7 days also reduced mRNA levels of IL-1β and TNF-α in rats (Viana et al., 2019).

We found reduction of IL-1β in trigeminal ganglia in the model of carrageenan-induced masseter inflammation, as well as decreased p38-MAPK activation in this neural structure in the model of formalin-induced nociception. Of interest, IL-1 β leads to nociceptor activation and contributes to pain hypersensitivity, which is a phenomenon described as depending on p38-MAPK activation in isolated dorsal root ganglion neurons (Binshtok et al., 2008). Therefore, despite the difference in models, our findings seem to corroborate, at least in part, the inhibitory effect of myrtenol in the p38-MAPK modulation in trigeminal ganglia after peripheral challenge with flogistic agents.

A limitation of our study is that we used only male mice, so we did not consider the gender variability, which is potentially important when analyzing the effect of drug candidates in models of nociception (Melchior et al., 2016).

## Conclusion

Our study demonstrated that treatment with myrtenol causes antinociceptive action due to inhibition of p38-MAPK activation in trigeminal ganglia and anti-inflammatory effect related to the reduction of IL-1β levels in trigeminal ganglia.

## Data Availability

The raw data supporting the conclusion of this article will be made available by the authors, without undue reservation.
